# Ovarian cyst torsion in a pre-menopausal woman causing intestinal obstruction: a case report

**DOI:** 10.11604/pamj.2023.45.93.38690

**Published:** 2023-06-21

**Authors:** Vasiliki-Kalouda Tsapadikou, Konstantinos Zacharis, Asimina-Paraskevi Barbarousi, Spyridon Chondros, Stavros Kravvaritis, Anastasia Fouka, Theodoros Charitos

**Affiliations:** 1 Department of Obstetrics and Gynaecology, General Hospital of Lamia, Lamia, Greece

**Keywords:** Intestinal obstruction, ovarian cyst, torsion, case report

## Abstract

Intestinal obstruction due to adnexal torsion is a rare complication that can be occurred during torsion of an ovarian cyst. A premenopausal woman presented to the emergency department with complaints of abdominal distension, abdominal pain, and obstipation for 2 days. An abdominal radiograph showed signs of large bowel partial obstruction. Hence admission to the surgical department was ordered. Due to deterioration of the patient, a gynaecological evaluation took place. Ultrasonography demonstrated a large ovarian cyst, which was also confirmed by an abdominal computed tomography scan and thus immediate laparotomy was decided. Abdominal hysterectomy with bilateral salpingo-oophorectomy was performed due to torsion of a giant ovarian cyst, which caused intestinal obstruction by compression. The post-operative course of the patient was uneventful. Ovarian torsion should not be eliminated from differential diagnosis when it comes to female patients with clinical presentation relevant to small and/or large bowel obstruction.

## Introduction

Ovarian torsion is caused by rotation of the ovary or adnexa with the vascular pedicle on its axes resulting in venous and arterial obstruction [1]. Torsion of the right adnexa occurs more commonly due to hypermobility of the right utero-ovarian ligament which is longer than the left. The majority of the patients present with abdominal pain, fever vomiting and/or nausea. These non-specific symptoms may lead to delayed diagnosis and physicians may face a diagnostic dilemma. In spite of being only 2.7% of acute gynaecological conditions [2], ovarian torsion requires immediate surgery. We hereby report a case of an ovarian cyst torsion causing partial intestinal obstruction.

## Patient and observation

**Patient information:** a 47-year-old woman presented to the emergency department with complaints of abdominal distension, abdominal pain and obstipation for 2 days. She also had a history of nausea but no vomiting. She had not visited a gynecologist for annual examination in the past 2 years due to the severe COVID-19 outbreak.

**Clinical findings:** on examination, she was well-built with blood pressure of 110/63 mmHg, pulse rate of 98 bpm and her body temperature was normal. She had mildly distended abdomen though was diffuse tenderness and a mass in her left iliac fossa and suprapubic region. Bowel sounds were present. A digital rectal examination revealed fecal staining in the rectum.

**Figure 1:**
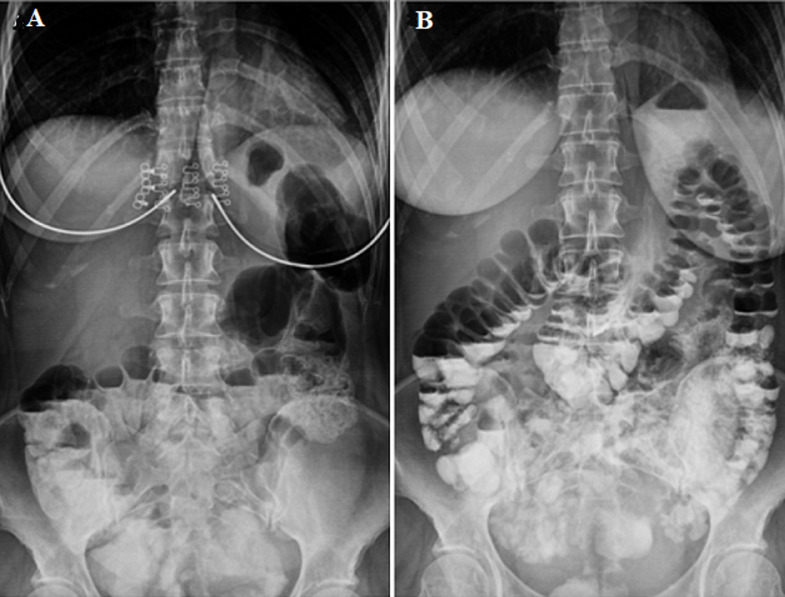
abdominal X-ray of the patient: A) on admission to the emergency department; B) after oral administration of gastrografin gastroenteral solution

**Timeline of current episode:** an abdominal radiograph showed signs of large bowel partial obstruction. The patient was given 30 ml of gastrografin gastroenteral solution; on the radiograph, the gastrografin appeared to be present in the rectum along with air-fluid levels (Figure 1).

**Diagnostic assessment:** routine blood investigations revealed normal leukocyte counts of about 8000 cells/mm^3^ and an elevated C-reactive protein level of 159 mg/L.

**Figure 2:**
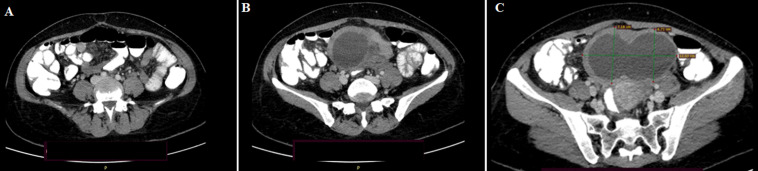
A,B,C) computerized tomography showing air-fluid levels in the small intestine and the size of the ovarian cyst

**Diagnosis:** admission in the surgical department was ordered, and the patient was treated over intestinal obstruction with intravenous fluids and antibiotics. Due to deterioration of the patient´s symptoms, a gynaecological evaluation took place. Both transabdominal and transvaginal ultrasonography demonstrated a large ovarian cyst of 11.8x6 cm with thick walls but no septation, which was also confirmed by an abdominal computed tomography scan that was carried out (Figure 2).

**Figure 3:**
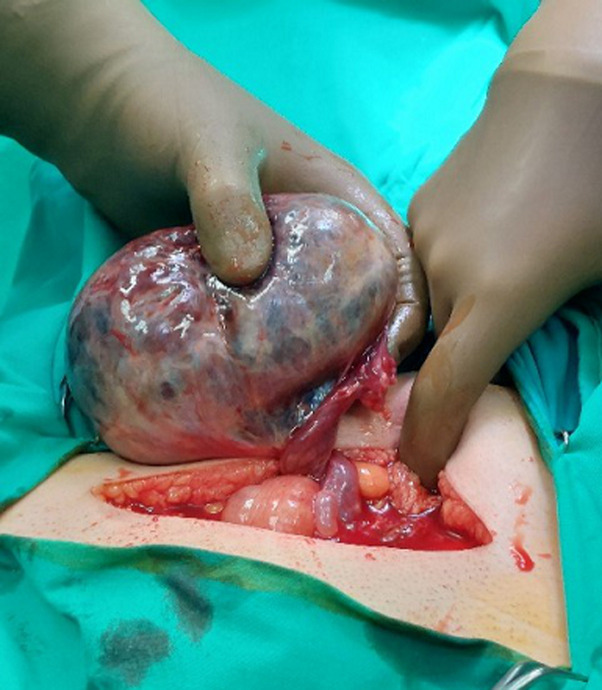
perioperative picture showing the torted stem of the cyst in situ

**Therapeutic interventions:** a decision for laparotomy was made, after informed consent. Intraoperative findings include a huge infracted, torted left ovarian cyst, adherent to the sigmoid hence causing partial intestinal obstruction by compression. The cyst was removed and an abdominal hysterectomy with bilateral salpingo-oophorectomy was carried out due to two big fibroids that were found during the transvaginal ultrasound (Figure 3).

**Follow-up and outcome of interventions:** post-operative ileus was settled on post-operative day 2, so oral feeding was started on day 4 and the patient was discharged after 5 days overall. Histopathology of the specimen revealed a follicular cyst with necrosis due to torsion measuring 12.5 cm.

**Patient perspective:** “I thought that the abdominal pain and the constipation was a digestive disease. I felt quite stressed when I heard about the computerized tomography (CT) findings. I should have never skipped my annual gynecologic examination, due to the pandemic. Fortunately, everything worked out in the end.”

**Informed consent:** written informed consent was obtained from the patient.

## Discussion

Ovarian cysts with a diameter of at least 10 cm may cause abdominal pain, swelling and vaginal bleeding [3]. Simple ovarian cysts are the most common non neoplastic adnexal masses among women of reproductive age [2,3]. Ovarian torsion can happen if an ovarian mass or cyst rotates the uteroovarian and the infundibulopelvic ligament and is rarer on the left side [1]. Intestinal obstruction due to ovarian torsion is a very occasional complication of ovarian cyst [4,5] and might be present through two mechanisms; either a giant ovarian mass may compress the bowel [2] or a loop of small and/or large bowel becomes adherent to the cyst and rotates with the torsion of the cyst [4]. Although ovarian torsion during pregnancy is relatively rare, adnexal torsion during pregnancy has been reported [1], as well as intestinal obstruction during pregnancy caused by bilateral ovarian cystic teratoma [6]. The clinical symptoms of ovarian torsion are nonspecific such as abdominal or pelvic pain, nausea and vomiting [1], which is similar to intestinal obstruction clinical appearance; the latest includes nausea, vomiting, bloating, crampy, colicky abdominal pain and minimal or complete absence of flatus and bowel movements [4].

## Conclusion

Ovarian torsion should not be eliminated from differential diagnosis when it comes to female patients with clinical presentation relevant to small and/or large bowel obstruction.
